# Effects of phosphodiesterase 4 inhibition on bleomycin-induced pulmonary fibrosis in mice

**DOI:** 10.1186/1471-2466-10-26

**Published:** 2010-05-05

**Authors:** Sergey Udalov, Rio Dumitrascu, Soni S Pullamsetti, Hamza M Al-tamari, Norbert Weissmann, Hossein A Ghofrani, Andreas Guenther, Robert Voswinckel, Werner Seeger, Friedrich Grimminger, Ralph T Schermuly

**Affiliations:** 1 University of Giessen, Department of Internal Medicine, Giessen, Germany; 2 Max-Planck-Institute for Heart and Lung Research, Department of Lung Development and Remodeling, Bad Nauheim, Germany

## Abstract

**Background:**

Pulmonary fibrosis (PF) is a group of devastating and largely irreversible diseases. Phosphodiesterase (PDE) 4 is involved in the processes of remodeling and inflammation, which play key role in tissue fibrosis. The aim of the study was, therefore, to investigate the effect of PDE4 inhibition in experimental model of PF.

**Methods:**

PF was induced in C57BL/6N mice by instillation of bleomycin. Pharmacological inhibition of PDE4 was achieved by using cilomilast, a selective PDE4 inhibitor. Changes in either lung inflammation or remodeling were evaluated at different stages of experimental PF. Lung inflammation was assessed by bronchoalveolar lavage fluid (BALF) differential cell count and reverse transcription quantitative polymerase chain reaction (RT-qPCR) for inflammatory cytokines. Changes in tissue remodeling were evaluated by pulmonary compliance measurement, quantified pathological examination, measurement of collagen deposition and RT-qPCR for late remodeling markers. Survival in all groups was analyzed as well.

**Results:**

PDE4 inhibition significantly reduced the total number of alveolar inflammatory cells in BALF of mice with bleomycin-induced PF at early fibrosis stage (days 4 and 7). Number of macrophages and lymphocytes, but not neutrophils, was significantly reduced as well. Treatment decreased lung tumor necrosis factor (TNF)-α mRNA level and increased mRNA level of interleukin (IL)-6 but did not influence IL-1β. At later stage (days 14 and 24) cilomilast improved lung function, which was shown by increase in lung compliance. It also lowered fibrosis degree, as was shown by quantified pathological examination of Hematoxilin-Eosin stained lung sections. Cilomilast had no significant effect on the expression of late remodeling markers such as transforming growth factor (TGF)-β1 and collagen type Ia1 (COL(I)α1). However, it tended to restore the level of lung collagen, assessed by SIRCOL assay and Masson's trichrome staining, and to improve the overall survival.

**Conclusions:**

Selective PDE4 inhibition suppresses early inflammatory stage and attenuates the late stage of experimental pulmonary fibrosis.

## Background

Pulmonary fibrosis represents a group of devastating and largely irreversible human interstitial lung diseases having only limited treatment options. The disease is characterized by chronic interstitial inflammation, abnormal function of interstitial fibroblasts and deposition of excessive amounts of collagen, altogether leading to severe tissue remodeling [1]. Pathological changes are accompanied by elevated expression of cytokines TNF-α, IL-1β, IL-6, growth factors and matrix metalloproteases (MMPs) [2-4]. The most common experimental model of human PF is bleomycin-induced PF in mice. It is characterized by inflammatory and remodeling stages, which allows studying different aspects of the disease [5].

Phosphodiesterases are a superfamily of enzymes that hydrolyze cAMP and/or cGMP and thereby regulate the intracellular levels of second messengers [6]. Members of PDE4 family (E.C. 3.1.4.17) are cAMP-specific PDEs composed of number of isoforms and are highly represented in the lung [7-10]. As a component of cAMP/protein kinase A (PKA) pathway PDE4 plays direct role in cell proliferation, differentiation and migration through regulation of the cAMP level [11-13]. Finally, PDE4 represents the major cAMP-hydrolyzing enzyme in monocytes, lymphocytes and neutrophils and its activation is required for inflammatory response [8,14,15].

For these reasons, PDE4 inhibitors were suggested for treatment of several lung diseases as new anti-inflammatory and anti-remodeling agents [16]. Our group has previously demonstrated that PDE3/4 inhibitor attenuates migration of pulmonary artery smooth muscle cells (PASMCs) *in vitro *and reverses pulmonary vascular remodeling *in vivo *[17]. PDE4 inhibitor cilomilast was also shown to suppress the release and activation of MMP-1 and MMP-9 from lung fibroblasts, which are known to be involved in PF progression [18]. Furthermore, cilomilast and other PDE4 inhibitors were demonstrated to inhibit lung TNF-α and TGF-β release, as well as neutrophil influx *in vivo *[19-21]. Finally, treatment of experimental chronic colitis with PDE4 inhibitor rolipram resulted in decreased collagen deposition as well as TNF-α and TGF-β content in the tissue [22].

In the present study we hypothesized that PDE4 inhibitors are able to modulate both inflammatory response and tissue remodeling. The aim of the study was, therefore, to investigate the effects of selective PDE4 inhibitor on different aspects of experimental PF *in vivo*.

## Methods

### Animals

Adult male 5-6 weeks-old C57BL/6N mice weighting 19-21 g were obtained from Charles River Laboratories (Germany). Animals were housed under room temperature and 12/12-hour light/dark cycle with free access to food and water. All experiments were performed in accordance with the National Institutes of Health Guidelines on the Use of Laboratory Animals. Both the University Animal Care Committee and the Federal Authorities for Animal Research of the Regierungspraesidium Giessen (Hessen, Germany) approved the study protocol.

### Bleomycin administration and treatment groups

At day 0 mice were given anesthesia with isofluran (Baxter, Germany) followed by random orotracheal instillation of bleomycin (Sigma, Germany) or sterile saline (0.9% NaCl) with the mouse nose kept pinched. Bleomycin dissolved in sterile saline was given at the dose of 2.8 units/kg. Animals were assigned to the following groups 1) "saline", 2) "bleo+ctrl" and 3) "bleo+cilo". "Saline" group received instillation of sterile saline at day 0 and was given vehicle alone (2% aqueous methylcellulose solution). Mice in "bleo+ctrl" group received instillation of bleomycin at day 0 and were given vehicle alone. Mice in "bleo+cilo" group received instillation of bleomycin at day 0 and were treated once a day with 50 mg/kg cilomilast (SB207499 or Ariflo^®^, [c-4-cyano-4-(3-cyclopentyloxy-4-methoxyphenyl)-r-l-cyclohexane carboxylic acid]) (Nycomed, Germany), suspended in vehicle. Solutions were administered *per os *via feeding needle, all in the same manner. Treatment in all groups started at day 0 and lasted till the end of experiment, i.e. for 4, 7, 14 or 24 days.

### Bronchoalveolar lavage fluid cell count

At days 4 and 7 after bleomycin instillation mice were euthanized by injecting a lethal dose of pentobarbital. Lungs were flushed three times with 0.5 ml ice cold PBS-EDTA, recovered fluid was centrifuged and cell pellet was re-suspended in 1 ml of ice-cold saline. Total cell count was performed using Neubauer counting chamber (depth 0.1 mm, 0.0025 mm^2^; Optik Labor, Germany). For differential cell count cells in constant volume of 0.2 ml of PBS were transferred to a glass slide with Cytospin-3 centrifuge (Shandon Scientific Ltd, UK) and stained with May Gruenwald/Giemsa. Numbers of macrophages, neutrophils and lymphocytes were determined by counting on light microscope (Q550IW; Leica, Germany) among 100 of total cells. These data were then extrapolated to number of cells per milliliter.

### Lung compliance and histological examination

At days 14 and 24 after bleomycin instillation mice were subjected to lung compliance measurement as described previously [23]. Briefly, animals were anesthetized with i.p. injection of ketamin/xylacinehydrochloride (Bayer, Germany). Trachea was canulated, mice were placed in the chamber and connected to the instrument. Volume of 0.3 ml and pressure of 3 kPa were set for calculating compliance as a ratio of volume to pressure (ml/kPa). Lungs of mice were isolated at the same time points. Four right lobes were inflated with 4.5% formaldehyde solution at constant pressure and fixated as described elsewhere. After dehydration in tissue processor lung lobes were individually embedded in paraffin, sectioned at 3 μm on microtome (all instruments from Leica, Germany), mounted on glass slides and stained either with Hematoxilin-Eosin or Masson's trichrome according to standard protocols. For histological assessment, Hematoxilin-Eosin-stained slides were scanned with light microscope (Leica, Germany) at 100× magnification yielding 50-100 images for each lobe (up to 300 per animal). Each of the images was reviewed and degree of fibrosis was assigned according to Ashcroft's fibrosis score system [24] with slight modifications: normal lung was referred to as score "0" while score "6" represented maximal degree of pathological changes.

### Collagen assay

Level of collagen in lung tissues was determined by SIRCOL collagen assay (Biocolor Ltd., UK) according to manufacturer's instructions. Briefly, left lung lobes were homogenized and collagen was solubilized in 0.5 M acetic acid. Extracts were incubated with Sirius red dye and absorbance was determined at 540 nm with spectrophotometer Infinite M200 (Tecan, Austria). Amount of collagen was expressed in μg/g of wet tissue.

### Survival analysis

Survival of mice for each treatment group was expressed in percent of animals left of original number at the specific time points of the experiment. Mice were followed up for 24 days.

### RNA isolation and cDNA synthesis

For RNA extraction left lung lobes were isolated and snap-frozen in liquid nitrogen. Tissues were homogenized with Precellys 24 homogenizer (Bertin Technologies, France) in 0.5 ml of TRIzol reagent (Invitrogen, USA) and RNA was extracted according to standard protocol. cDNA synthesis was carried out with ImProm-II™ Reverse Transcription System (Promega, USA) according to manufacturer's instruction.

### Real-time polymerase chain reaction

For quantitative real-time PCR analysis, cDNA was amplified with Platinum^® ^SYBR^® ^Green qPCR SuperMix-UDG mix (Invitrogen, USA). Specific PCR primers (Metabion, Germany), designed to anneal to adjacent exons to exclude possible amplification from genomic DNA, were: mouse β-actin 5'-CTCTAGACTTCGAGCAGGAGATG-3' (forward) and 5'-CACTGTGTTGGCATAGAGGTCTT-3' (reverse); mouse TNF-α 5'-GCCTATGTCTCAGCCTCTTCTC-3' (forward) and 5'-CACTTGGTGGTTTGCTACGA-3' (reverse); mouse IL-1β 5'-GAGCACCTTCTTTTCCTTCATCT-3' (forward) and 5'-GATATTCTGTCCATTGAGGTGGA-3' (reverse); mouse IL6 5'-TCAATTCCAGAAACCGCTATGAA-3' (forward) and 5'-CACCAGCATCAGTCCCAAGAA-3' (reverse); mouse COL(I)a1 5'-AGCTTTGTGGACCTCCGGCT-3' (forward) and 5'-ACACAGCCGTGCCATTGTGG-3' (reverse); mouse TGF-β1 5'-AACCCCCATTGCTGTCCCGT-3' (forward) and 5'-CCTTGGTTCAGCCACTGCCG-3' (reverse). Quantitative real-time PCR was carried out in Srtratagene Mx3000P™ qPCR system (Stratagene, USA) and data were analyzed with accompanying software. The instrument was programmed as follows: denaturation, 95°C for 10 min; 40 cycles with denaturation at 95°C for 30 s, annealing at 58-60°C for 30 s and extension at 72°C for 30 s. To ensure that specific single product is generated dissociation curves were evaluated. Relative expression levels were calculated as ΔCt values by normalizing Ct values of target genes to Ct values of β-actin as previously described [17].

### Statistical analysis

All data are presented as means +/- SEM. The differences among groups were assessed by one-way Analysis of Variance (ANOVA) test and Student-Newman-Keuls post-test for multiple comparisons. A *p*-value less than 0.05 was considered statistically significant.

## Results

### Effect of PDE4 inhibition on alveolar inflammatory cells content

**Figure 1:**
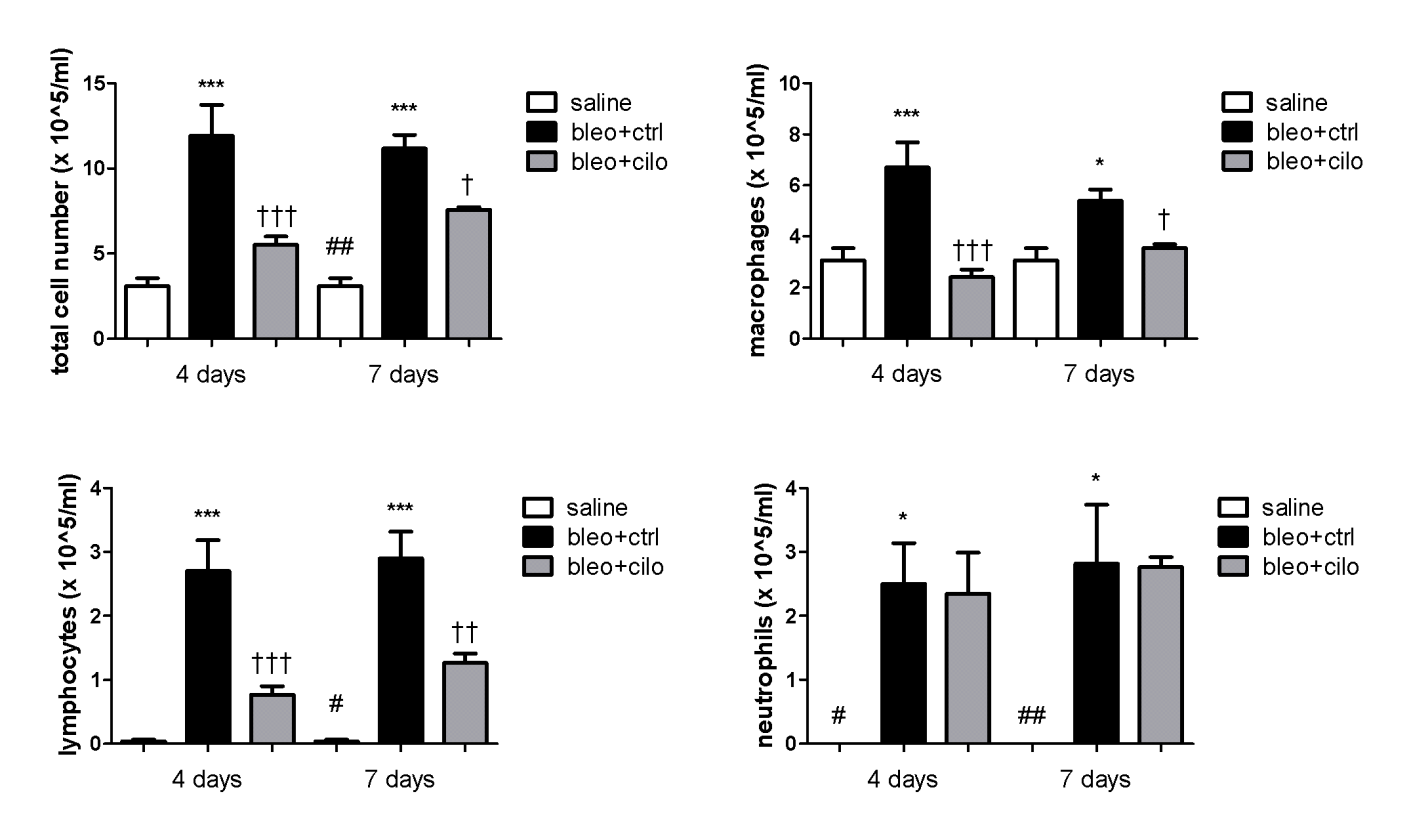
**PDE4 inhibition suppresses alveolar inflammation at early stage fibrosis**. Total cell number, number of macrophages, lymphocytes and neutrophils in bronchoalveolar lavage fluid (BALF) of healthy controls treated with vehicle ("saline") and in mice suffering from fibrosis and treated either with vehicle ("bleo+ctrl") or cilomilast ("bleo+cilo") at days 4 and 7 after bleomycin instillation. Values are presented as means ± SEM, n = 6. * saline vs. bleo+ctrl (*p < 0.05, ***p < 0.001), † bleo+ctrl vs. bleo+cilo († p < 0.05, †† p < 0.01, ††† p < 0.001), # saline vs. bleo+cilo (# p < 0.05, ## p < 0.01).

To assess the effect of cilomilast on lung inflammation, BALF was collected at early stage of bleomycin-induced fibrosis from healthy mice treated with vehicle and mice that received bleomycin and treated either with cilomilast or vehicle. Total number of alveolar inflammatory cells was dramatically increased by instillation of bleomycin (Fig. 1). In contrast, number of cells was significantly lower in groups that received cilomilast, both at 4 and 7 days (p < 0.001 and p < 0.05 respectively).

To further evaluate the action of cilomilast on different inflammatory cell types differential cell count was performed (Fig. 1). As expected, all cell types were highly present in alveolar space after bleomycin instillation, with the highest increase in number of lymphocytes and neutrophils. Number of macrophages and lymphocytes was significantly decreased by cilomilast both at 4 days (p < 0.001 for both cell types) and 7 days (p < 0.05 and p < 0.01 for macrophages and lymphocytes respectively). Number of neutrophils, however, remained unchanged.

### Effect of PDE4 inhibition on lung inflammatory markers

**Figure 2:**
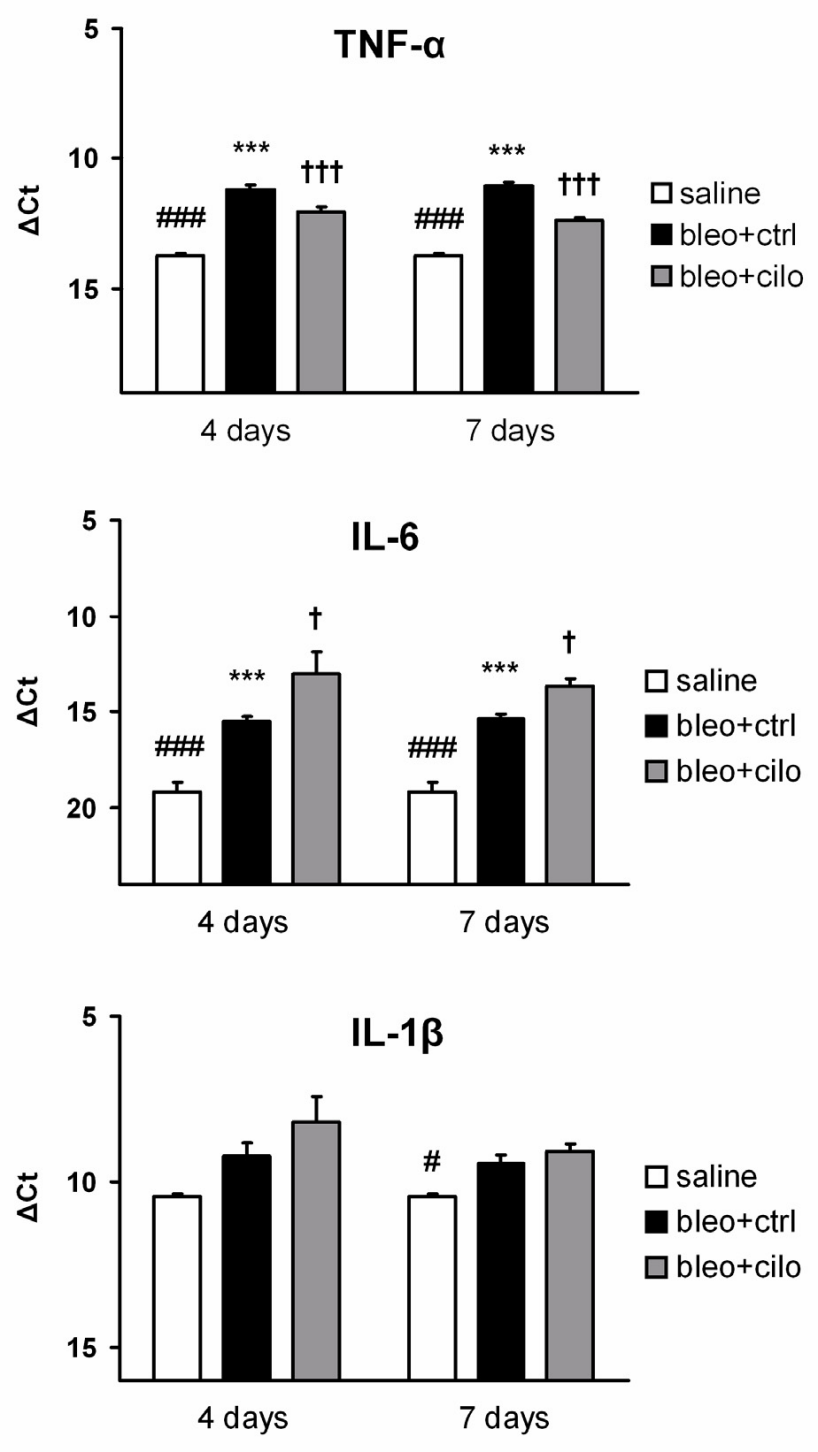
**Effect of PDE4 inhibition on lung inflammatory markers at early stage fibrosis**. TNF-α, IL-6 and IL-1β mRNA levels in healthy controls treated with vehicle ("saline") and in mice suffering from fibrosis and treated either with vehicle ("bleo+ctrl") or cilomilast ("bleo+cilo") at days 4 and 7 after bleomycin administration. RT-qPCR data are normalized to β-actin expression and presented as ΔCt values ± SEM, n = 6. * saline vs. bleo+ctrl (***p < 0.001), † bleo+ctrl vs. bleo+cilo († p < 0.05, ††† p < 0.001), # saline vs. bleo+cilo (# p < 0.05, ### p < 0.001).

To evaluate the expression of major inflammatory markers after cilomilast treatment, lung homogenate RT-qPCR was carried out at the same time points as for BALF cell count. At 4 and 7 days after bleomycin instillation lung expression of TNF-α, IL-1β and IL-6 was significantly elevated compared to animals that received saline (Fig. 2). Treatment with cilomilast significantly lowered TNF-α mRNA level (p < 0.001 at 4 and 7 days) and increased IL-6 mRNA level (p < 0.05 at 4 and 7 days) compared to mice treated with vehicle. No significant change, however, was observed in the level of IL-1β.

### Effect of PDE4 inhibition on lung function and pathology

**Figure 3:**
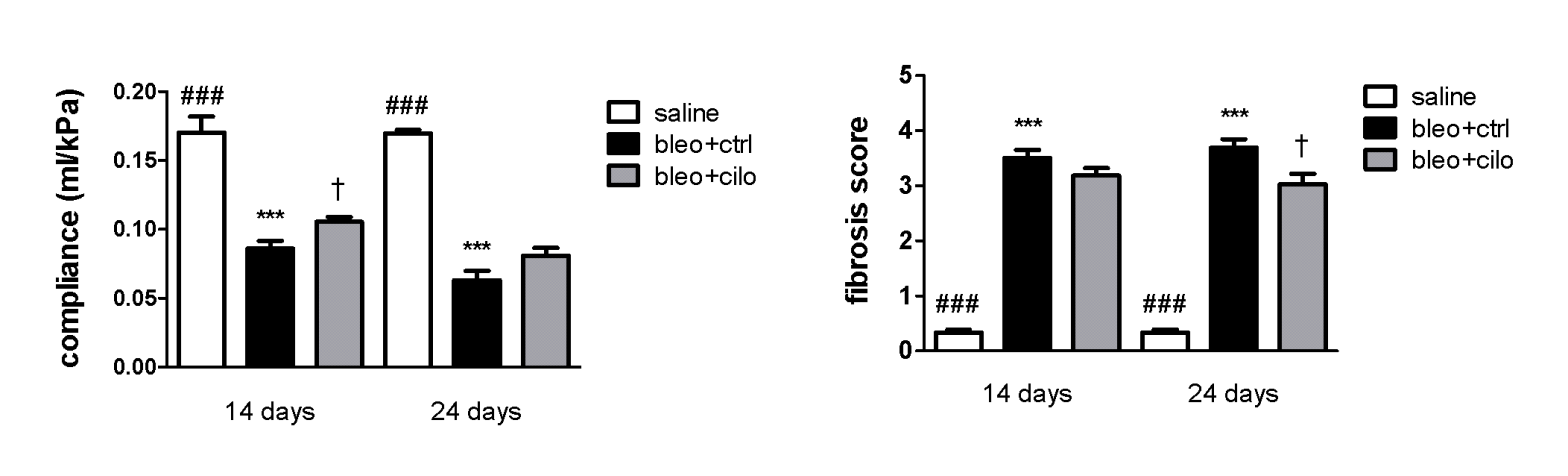
**PDE4 inhibition improves lung function and attenuates tissue remodeling at late stage fibrosis**. Lung compliance and fibrosis scores in healthy controls treated with vehicle ("saline") and in mice suffering from fibrosis and treated either with vehicle ("bleo+ctrl") or cilomilast ("bleo+cilo") at days 14 and 24 after bleomycin administration. Values are presented as means ± SEM, n = 9. * saline vs. bleo+ctrl (***p < 0.001), † bleo+ctrl vs. bleo+cilo († p < 0.05), # saline vs. bleo+cilo (### p < 0.001).

To examine the effect of cilomilast on tissue remodeling at late stage fibrosis, lung compliance and pathological changes were evaluated in animals treated either with cilomilast or vehicle, as well as in mice received instillation of saline and treated with vehicle. Pulmonary compliance (Fig. 3) was significantly lower in mice with bleomycin-induced fibrosis both at 14 and 24 days, which shows a typical decrease in elasticity of the lung tissue. Treatment with cilomilast partially restored impaired lung function with improvement being significant at 14 days (p < 0.05), compared to mice treated with vehicle alone.

Similarly, pathological changes estimated by means of microscopy followed by scoring (Fig. 3) evidenced significant distortion of lung architecture in mice with bleomycin-induced fibrosis. Degree of fibrosis was lower in lungs of animals treated with PDE4 inhibitor compared to ones treated with vehicle only, reaching significance at day 24 (p < 0.05).

**Figure 4:**
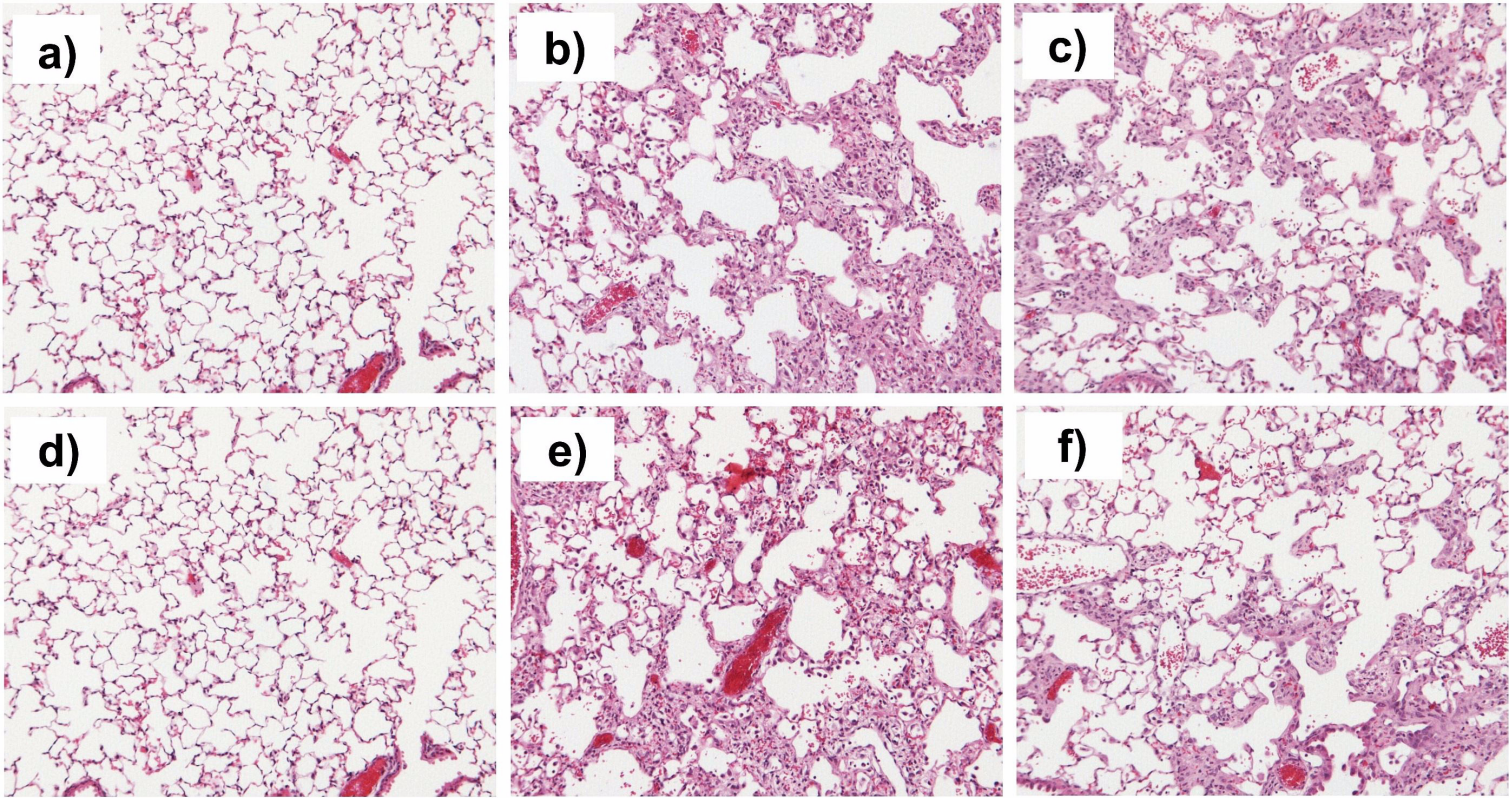
**PDE4 inhibition attenuates tissue remodeling at late stage fibrosis**. Representative images of lungs of healthy controls treated with vehicle (a, d) and of mice suffering from fibrosis and treated either with vehicle (b, e) or cilomilast (c, f) at days 14 (a, b, c) and 24 (d, e, f) after bleomycin administration. Hematoxilin-Eosin staining, magnification ×100.

Representative images of lung sections stained with Hematoxilin-Eosin (Fig. 4) demonstrate the degree of mentioned pathological changes quantified by fibrosis scoring.

### Effect of PDE4 inhibition on lung collagen content and remodeling markers

**Figure 5:**
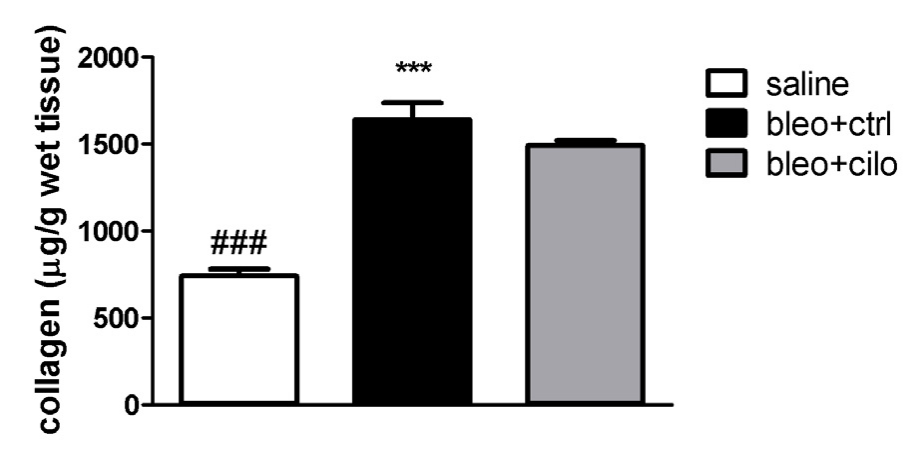
**Effect of PDE4 inhibition on lung collagen content at late stage fibrosis**. SIRCOL assay for the lungs of healthy controls treated with vehicle ("saline") and in mice suffering from fibrosis and treated either with vehicle ("bleo+ctrl") or cilomilast ("bleo+cilo") at day 24 after bleomycin administration. Values are presented as means ± SEM, n = 4. * saline vs. bleo+ctrl (***p < 0.001), # saline vs. bleo+cilo (### p < 0.001).

Collagen content in the lungs was estimated by SIRCOL assay, Masson's trichrome staining and RT-qPCR for COL(I)α1 at day 24 or at day 14 and 24 after bleomycin administration in all treatment groups. Total collagen assay (μg/g tissue) showed significant 2-fold increase among mice received bleomycin (Fig. 5), which was confirmed by Masson's trichrome staining (Additional file 1). Treatment with cilomilast tended to reduce collagen content in the lungs, although the effect was rather moderate. In contrast, virtually no effects were observed at mRNA level of COL(I)α1 at particularly at day 24 (Additional file 2). Similarly to collagen, cilomilast had no significant effect on mRNA level of TGF-β1 (Additional file 3).

### Effect of PDE4 inhibition on survival

**Figure 6:**
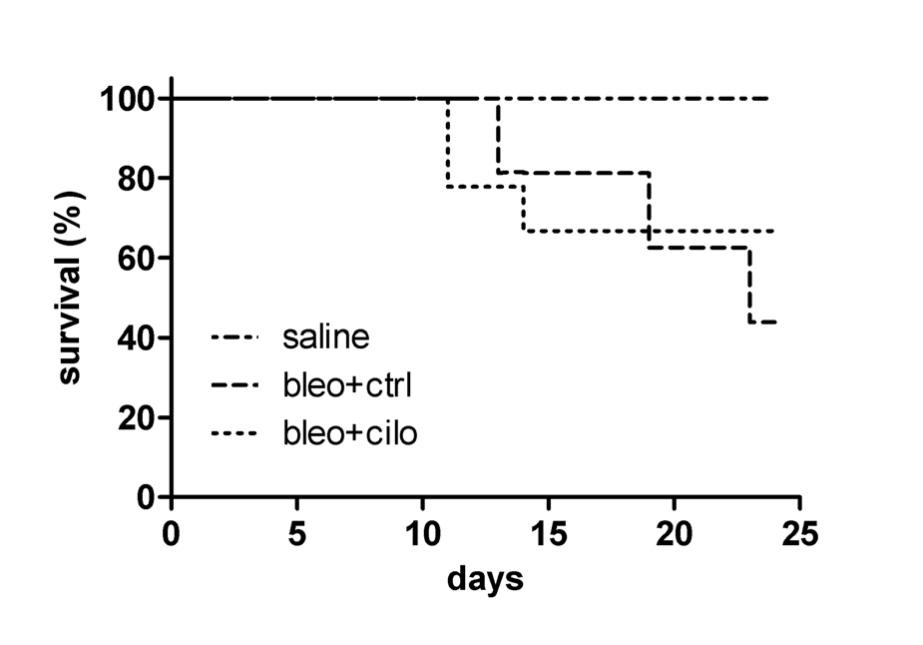
**Effect of PDE4 inhibition on survival**. Healthy controls treated with vehicle ("saline") and mice suffering from fibrosis and treated either with vehicle ("bleo+ctrl") or cilomilast ("bleo+cilo") followed up for 24 days after bleomycin instillation. Kaplan-Meier curves, n = 9.

General effect of cilomilast on the course of experimental PF was evaluated by survival analysis (Fig. 6). Inhibition of PDE4 had beneficial effect on survival as was seen at the end of experiment (day 24) in the group that received cilomilast compared to one treated with vehicle alone. No mortalities were observed in the group that received sterile saline.

## Discussion

In the present study we have demonstrated beneficial anti-inflammatory effects of selective PDE4 inhibitor cilomilast at inflammatory stage of experimental PF, including reduction in BALF cell numbers, suppression of TNF-α and stimulation of IL-6 expression. We have also demonstrated improvements in lung function and pathological changes at later fibrosis stages. Finally, we have showed that treatment with PDE4 inhibitor tends to reduce lung collagen content and to improve the overall survival of the animals with bleomycin-induced PF.

Both human PF and bleomycin-induced PF in mice are characterized by chronic interstitial inflammation [1,5]. Given that PDE4 is the major cAMP-hydrolyzing enzyme in inflammatory cells and that it is necessary for developing of inflammatory response [8,14,15,25], several studies showed beneficial effect of PDE4 inhibitors on such inflammatory diseases as asthma and chronic obstructive pulmonary disease (COPD) [16]. Thus, we suggested positive effect of PDE4 inhibition on inflammatory component of PF.

Indeed, cilomilast was the most potent at early stage (days 4 and 7) of bleomycin-induced PF, when inflammation is the major characteristic of the pathological process [5]. Total number of alveolar inflammatory cells in BALF of treated mice was significantly reduced, as well as number of macrophages and lymphocytes. These results are consistent with the fact that PDE4 expression is induced by inflammatory stimulus and that it mediates activation and proliferation of T-cells and function of macrophages [25-28]. In turn, macrophages represent the major inflammatory cell type in alveolus, thereby strongly influencing total cell count values [29,30].

Neutrophils also play important role in pathological tissue remodeling damaging the lung parenchyma by proteolytic enzymes. Indeed, IPF patients have higher numbers of neutrophils and higher concentrations of granule enzymes, such as neutrophil elastase, myeloperoxydase, collagenase and lactoferrin in BALF, plasma and lung tissue [30,31]. Ariga et al. described direct involvement of PDE4 into neutrophil recruitment and chemotaxis [32] and Corbel et al. showed a decrease in neutrophils release by selective PDE4 inhibitor piclamilast in a murine model of LPS-induced acute lung inflammation [21]. However, we could not observe the significant suppression of neutrophil influx by cilomilast in our experimental setup. This inconsistency can be explained by early (hours) time-points used in acute lung inflammation experiments. The time-points used in the present work (4 and 7 days) were chosen to more closely mimic the inflammatory component of PF. But at the same time they are known to correspond to the peak in the neutrophil influx, therefore making it more difficult to achieve the significant improvement [33]. Another explanation might be the differential ability of the compounds to affect particular cell types and release of mediators. For instance, one study showed differential effect of several PDE4 inhibitors on neutrophils and TNF-a release, indeed revealing some limitations of cilomilast [20].

To find if general suppression of inflammatory cells release was also reflected in lung inflammatory cytokines expression, expression of TNF-α, IL-1β and IL-6 was analyzed at the same time points as the cell count experiments were done. These markers are upregulated in the lungs of both humans and mice with PF and are the canonical cytokines expressed at first days 4-7 of experimental PF [2-5]. We demonstrated significantly lower TNF-α expression in the lungs of mice treated with cilomilast compared to those received placebo. Taking into account lower numbers of BALF macrophages after cilomilast treatment it was expected to see downregulation of TNF-α as macrophages represent one of the major sources of this cytokine [2]. Similar effects of cilomilast were also shown by other authors [19]. In addition, significantly higher expression of IL-6 was observed in cilomilast-treated animals, compared to controls. This cytokine is known to exert anti-inflammatory effects and its exogenous administration was shown to decrease BALF cell recruitment, macrophage-mediated TNF-a production and lung hydroxyproline content in experimental pneumonitis in mice [34]. We suggest, therefore, that increase in IL-6 expression caused by cilomilast accompanies the general suppression of inflammatory cell influx and TNF-α content in the lung. Interestingly, cilomilast did not change lung expression of IL-1β. However, it was previously reported that PDE4 inhibitors have little or no effect on production of this cytokine [35]. Taken together, we suggest that PDE4 inhibition suppresses lung inflammation via modulation of TNF-α and IL-6.

Besides inflammation, PF is characterized by tissue remodeling and accumulation of extracellular matrix components. This ultimately results in impairment of gas exchange due to thickened interstitium and in worsening of lung mechanical properties due to increasing stiffness of the tissue [1]. As expected, decreased pulmonary compliance, higher fibrosis degree and higher lung collagen level were observed at days 14 and 24 in mice that received instillation of bleomycin. Progression of fibrosis is illustrated by lower compliance and higher fibrosis score at day 24 compared to day 14. Typical manifestations of bleomycin-induced PF, such as patchy pattern and interstitial inflammation were also observed. In turn, animals that received cilomilast demonstrated higher lung compliance and lower fibrosis score. As the infiltration of inflammatory cells into the interstitium might also contribute to impairment of the lung function we believe that the significant improvement in compliance at day 14 results from more effective suppression of interstitial inflammation at this time point compared to late remodeling stage at day 24. Treatment with PDE4 inhibitor also tended to reduce lung collagen accumulation, as was shown by total collagen assay and Masson's trichrome staining, particularly at day 14 after bleomycin administration. In contrast, no significant effects were seen at mRNA level of COL(I)α1, which might result from contribution of other collagen types' expression. Similarly, no effect of cilomilast treatment on TGF-β1 expression was observed.

Based on our observations and the results of other authors we suppose that inhibition of PDE4 affects both general aspects of PF, namely inflammation and tissue remodeling itself. At first, PDE4 inhibition suppresses tissue fibrosis by partial elimination of pro-fibrotic environment, for instance by suppression of inflammatory cells infiltration, downregulation of TNF-α and stimulation of IL-6 expression shown in the present work. TNF-α secreted by macrophages [2,36] is a direct mitogen for lung fibroblasts [37] and its inhibition can be alone sufficient to attenuate PF [38]. Moreover, it was shown that PDE4 itself is necessary for TNF-α production and development of inflammatory response [15,25]. Secondly, there are evidences implying that PDE4 inhibitors are also able to act through inflammation-independent way. For instance, it was repetitively shown that elevation of cAMP level results in inhibition of lung fibroblast proliferation, migration, transition to myofibroblasts and collagen production [11-13]. It was also shown that PKA can directly inhibit Raf thereby affecting the RAS/RAF/MEK/ERK pathway. Details of this interaction are not fully understood, however at least three possible mechanisms are suggested [39]. Our group has also previously demonstrated that cAMP raised by PDE3/4 inhibitor tolafentrine inhibits enhanced migration of PASMCs derived from vessels of rats suffering from pulmonary hypertension [17]. All together these data suggest that the effects observed in present study might be accounted to several independent actions of the PDE4 inhibitor, affecting both inflammatory process and the effector cells at the site of ongoing fibrosis.

## Conclusions

PDE4 inhibition by cilomilast attenuates bleomycin-induced pulmonary fibrosis in mice. Primarily, cilomilast exerts its beneficial effects via reduction of inflammatory response, although it does not significantly affect neutrophils release. Cilomilast treatment also moderately affects tissue remodeling at late fibrosis stage. This seems to be the consequence of its anti-inflammatory action, although direct effect on tissue remodeling via inflammation-independent mechanism is highly possible.

## Competing interests

SU received a doctoral stipendium from Altana Pharma AG. RTS received lecture fees from Encysive and Bayer-Schering and research grants from Ergonex, Encysive, Actelion and Bayer-Schering.

## Authors' contributions

SU carried out experimental work, data analysis and manuscript drafting. RD assisted in animal experiments and BALF cell count. SSP and RTS assisted in data analysis, participated in study design and reviewed the manuscript. HMA assisted in manuscript revision. AG, NW and HAG participated in study design. RV, FG and WS participated in study coordination. All authors read and approved the manuscript.

## Pre-publication history

The pre-publication history for this paper can be accessed here:

http://www.biomedcentral.com/1471-2466/10/26/prepub

## Supplementary Material

Additional file 1 **Effect of PDE4 inhibition on lung collagen deposition at late stage fibrosis**. Representative images of lungs of healthy controls (a, d) and of mice suffering from fibrosis and treated either with vehicle (b, e) or cilomilast (c, f) at days 14 (a, b, c) and 24 (d, e, f) after bleomycin administration. Masson's trichrome staining, magnification ×100. Click here for file

Additional file 2 **Effect of PDE4 inhibition on lung collagen expression at late stage fibrosis**. mRNA levels of COL(I)α1 in healthy controls ("saline") and in mice suffering from fibrosis and treated either with vehicle ("bleo+ctrl") or cilomilast ("bleo+cilo") at days 14 and 24 after bleomycin administration. RT-qPCR data are normalized to β-actin expression and presented as ΔCt values ± SEM, n = 4. Click here for file

Additional file 3 **Effect of PDE4 inhibition on late stage fibrosis**. mRNA levels of TGF-β1 in healthy controls ("saline") and in mice suffering from fibrosis and treated either with vehicle ("bleo+ctrl") or cilomilast ("bleo+cilo") at days 14 and 24 after bleomycin administration. RT-qPCR data are normalized to β-actin expression and presented as ΔCt values ± SEM, n = 4. Click here for file
