# Correction to: Effects of phosphodiesterase 4 inhibition on bleomycin-induced pulmonary fibrosis in mice

**DOI:** 10.1186/s12890-022-01876-5

**Published:** 2022-03-29

**Authors:** Sergey Udalov, Rio Dumitrascu, Soni Savai Pullamsetti, Hamza M. Al-tamari, Norbert Weissmann, Hossein Ardeschir Ghofrani, Andreas Guenther, Robert Voswinckel, Werner Seeger, Friedrich Grimminger, Ralph T. Schermuly

**Affiliations:** 1 Department of Internal Medicine, University of Giessen, Giessen, Germany; 2 Department of Lung Development and Remodeling, Max-Planck-Institute for Heart and Lung Research, Bad Nauheim, Germany

## Correction to: BMC Pulmonary Medicine 2010, 10:26 https://doi.org/10.1186/1471-2466-10-26

Following publication of the original article [1], it was brought to the authors’ attention that a representative image had been duplicated in Fig. 4 (panels A and D).

**Fig. 4:**
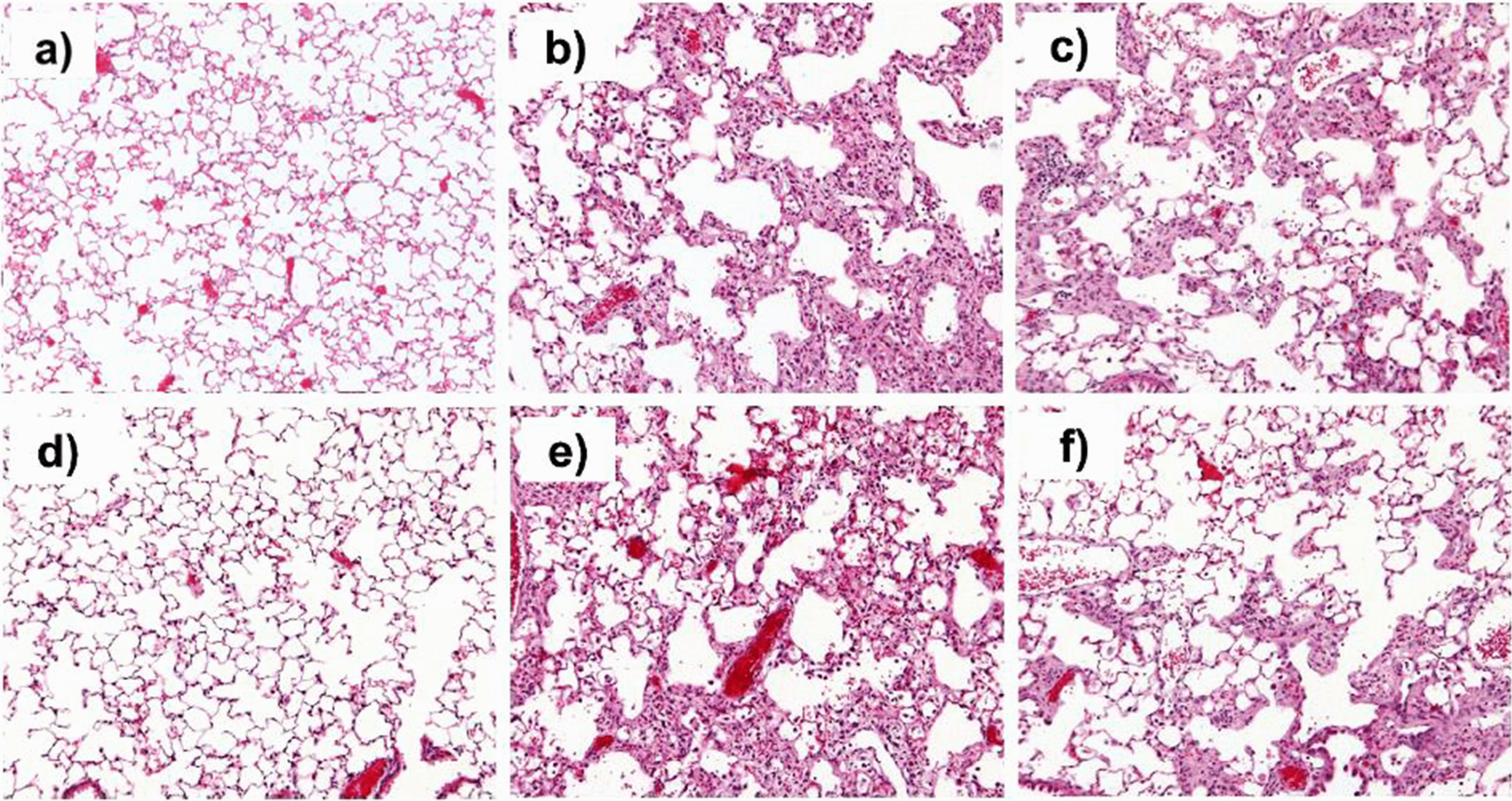
PDE4 inhibition attenuates tissue remodeling at late stage fibrosis. Representative images of lungs of healthy controls treated with vehicle (**a**, **d**) and of mice suffering from fibrosis and treated either with vehicle (**b**, **e**) or cilomilast (**c**, **f**) at days 14 (**a**, **b**, **c**) and 24 (**d**, **e**, **f**) after bleomycin administration. Hematoxilin-Eosin staining, magnification ×100

The figure has been corrected in the published article and the corrected figure can be seen below. This correction does not affect the results or conclusion.

The authors apologize for any inconvenience caused.
