# Supplementary figures and images for: Effects of phosphodiesterase 4 inhibition on bleomycin-induced pulmonary fibrosis in mice
Source: BMC Pulm Med. 2010 May 5;10:26. doi: 10.1186/1471-2466-10-26 (PMC2881047; doi:10.1186/1471-2466-10-26)

## Additional file 1

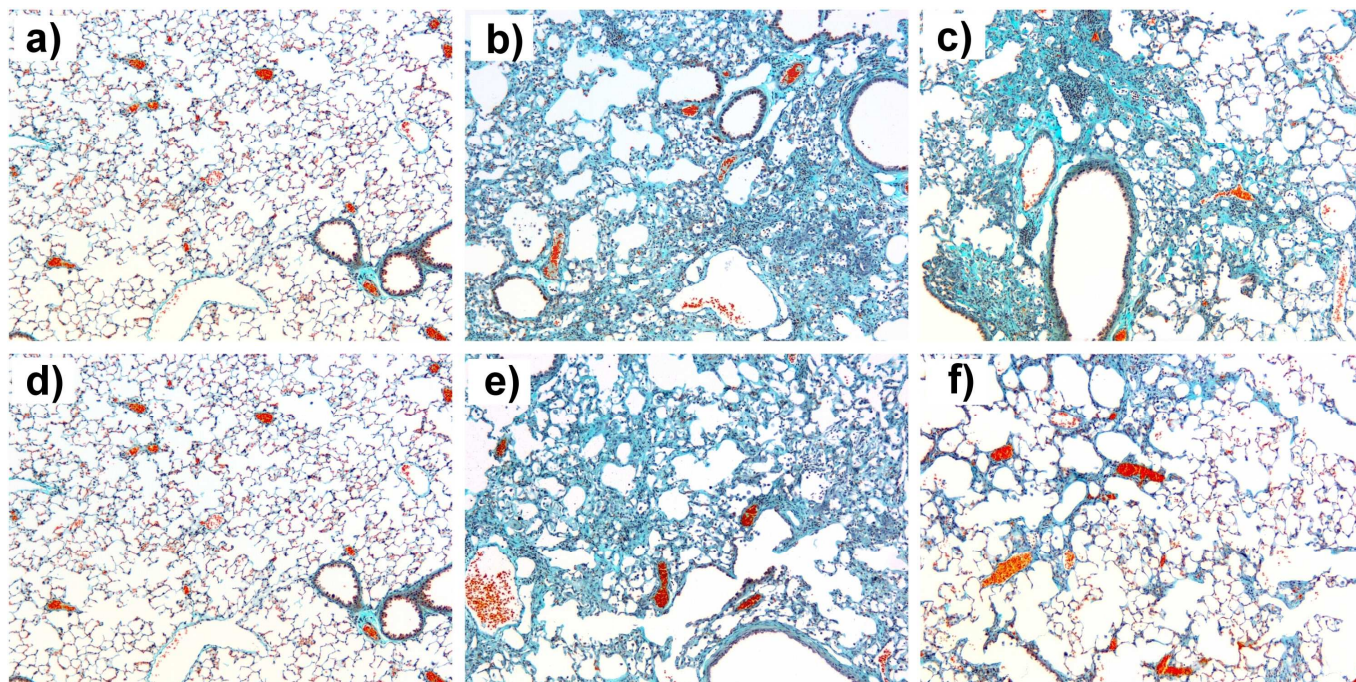

Supplement: Additional file 1 — Effect of PDE4 inhibition on lung collagen deposition at late stage fibrosis. Representative images of lungs of healthy controls (a, d) and of mice suffering from fibrosis and treated either with vehicle (b, e) or cilomilast (c, f) at days 14 (a, b, c) and 24 (d, e, f) after bleomycin administration. Masson's trichrome staining, magnification ×100. [file 1471-2466-10-26-S1.PDF]

## Additional file 2

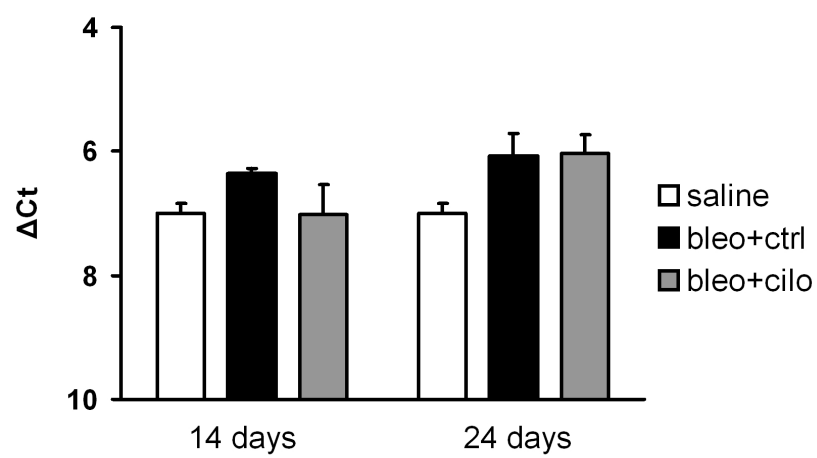

Supplement: Additional file 2 — Effect of PDE4 inhibition on lung collagen expression at late stage fibrosis. mRNA levels of COL(I)α1 in healthy controls ("saline") and in mice suffering from fibrosis and treated either with vehicle ("bleo+ctrl") or cilomilast ("bleo+cilo") at days 14 and 24 after bleomycin administration. RT-qPCR data are normalized to β-actin expression and presented as ΔCt values ± SEM, n = 4. [file 1471-2466-10-26-S2.PDF]

### Additional file 3

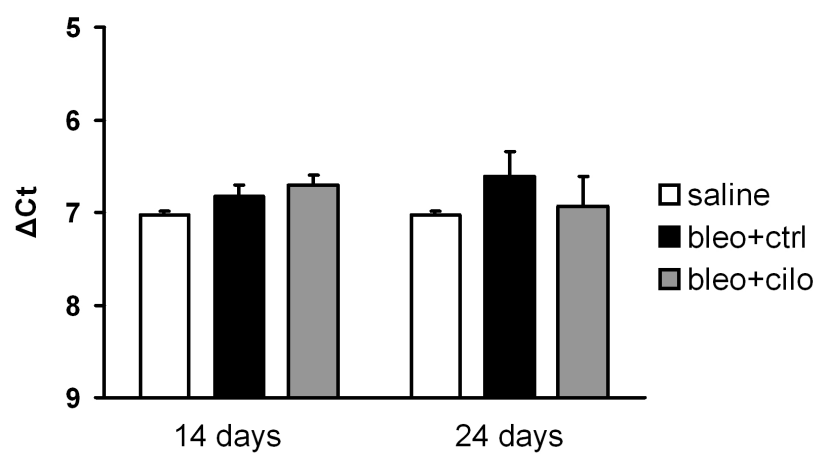

Supplement: Additional file 3 — Effect of PDE4 inhibition on late stage fibrosis. mRNA levels of TGF-β1 in healthy controls ("saline") and in mice suffering from fibrosis and treated either with vehicle ("bleo+ctrl") or cilomilast ("bleo+cilo") at days 14 and 24 after bleomycin administration. RT-qPCR data are normalized to β-actin expression and presented as ΔCt values ± SEM, n = 4. [file 1471-2466-10-26-S3.PDF]
